# Identification of two GH18 chitinase family genes and their use as targets for detection of the crayfish-plague oomycete *Aphanomyces astaci*

**DOI:** 10.1186/1471-2180-9-184

**Published:** 2009-08-31

**Authors:** Gerald Hochwimmer, Reinhard Tober, Renè Bibars-Reiter, Elisabeth Licek, Ralf Steinborn

**Affiliations:** 1 Institute of Bacteriology, Mycology and Hygiene, University of Veterinary Medicine, Veterinaerplatz 1, 1210 Vienna, Austria; 2 Vetomics Core Facility, University of Veterinary Medicine, Veterinaerplatz 1, 1210 Vienna, Austria; 3 Department of Biochemistry, University of Vienna, Dr.-Bohr-Gasse 9, 1030 Vienna, Austria; 4 Clinic for Avian, Reptile and Fish Medicine, University of Veterinary Medicine, Veterinaerplatz 1, 1210 Vienna, Austria

## Abstract

**Background:**

The oomycete *Aphanomyces astaci *is regarded as the causative agent of crayfish plague and represents an evident hazard for European crayfish species. Native crayfish populations infected with this pathogen suffer up to 100% mortality. The existence of multiple transmission paths necessitates the development of a reliable, robust and efficient test to detect the pathogen. Currently, *A. astaci *is diagnosed by a PCR-based assay that suffers from cross-reactivity to other species. We developed an alternative closed-tube assay for *A. astaci*, which achieves robustness through simultaneous amplification of multiple functionally constrained genes.

**Results:**

Two novel constitutively expressed members of the glycosyl hydrolase (GH18) gene family of chitinases were isolated from the *A. astaci *strain Gb04. The primary amino acid sequence of these chitinase genes, termed *CHI2 *and *CHI3*, is composed of an N-terminal signal peptide directing the post-translational transport of the protein into the extracellular space, the catalytic GH18 domain, a proline-, serine-, and threonine-rich domain and a C-terminal cysteine-rich putative chitin-binding site. The *A. astaci *mycelium grown in a pepton-glucose medium showed significant temporal changes in steady-state *CHI2 *and *CHI3 *mRNA amounts indicating functional constraint. Their different temporal occurrence with maxima at 48 and 24 hours of incubation for *CHI2 *and *CHI3*, respectively, is in accordance with the multifunctionality of GH18 family members. To identify *A. astaci*-specific primer target sites in these novel genes, we determined the partial sequence homologs in the related oomycetes *A. frigidophilus*, *A. invadans*, *A. helicoides*, *A. laevis*, *A. repetans*, *Achlya racemosa*, *Leptolegnia caudata*, and *Saprolegnia parasitica*, as well as in the relevant fungi *Fusarium solani *and *Trichosporon cutaneum*. An *A. astaci*-specific primer pair targeting the novel genes *CHI2 *and *CHI3 *as well as *CHI1 *- a third GH18 family member - was multiplexed with primers targeting the *5.8S rRNA *used as an endogenous control. A species was typed unambiguously as *A. astaci *if two peaks were concomitantly detected by melting curve analysis (MCA). For sensitive detection of the pathogen, but also for quantification of agent levels in susceptible crayfish and carrier crayfish, a TaqMan-probe based real-time PCR (qPCR) assay was developed. It targets the same chitinase genes and allows quantification down to 25 target sequences.

**Conclusion:**

The simultaneous qualitative detection of multiple sequences by qPCR/MCA represents a promising approach to detect species with elevated levels of genetic variation and/or limited available sequence information. The homogenous closed-tube format, reduced detection time, higher specificity, and the considerably reduced chance of false negative detection achieved by targeting multiple genes (*CHI1*, *CHI2*, *CHI3*, and the endogenous control) at least two of which are subject to high functional constraint, are the major advantages of this multiplex assay compared to other diagnostic methods.

Sensitive quantification achieved with TaqMan qPCR facilitates to monitor infection status and pathogen distribution in different tissues and can help prevent disease transmission.

## Background

Oomycetes are a group of filamentous, unicellular heterokonts. They are fungus-like in their growth form, adsorptive and parasitic lifestyles and formation of spores, but are relatively closely related to photosynthetic algae such as brown algae and diatoms [1]. Among oomycetes, also known as water molds, there are economically important pathogens that comprise severe pests, like *Phytophthora infestans *[2,3] causing potato late blight, *A. euteiches *causing seedling blight or legumes root rot [4], *A. astaci *[5], - the causative agent of crayfish plague, and several fish pathogens from the genera *Aphanomyces *[6], *Achlya *and *Saprolegnia *[7]. There is also at least one species with zoonotic potential, namely *Pythium insidiosum *- the etiologic agent of the human disease pythiosis insidiosii, which can be life-threatening [8]. The oomycetes *A. astaci *and *Phytophthora cinnamomi *are listed among the world's 100 worst invasive species (Global Invasive Species Database: http://www.issg.org/database, alphabetical list as of November 2008).

The crayfish plague, representing the most severe disease among Asian, Australian, and European crayfish species, is caused by the oomycete *A. astaci *(*Saprolegniales*, *Oomycetes*). Crayfish plague-associated die-offs in Austrian waters were first reported in 1879 [9] and in the 1920s [10], and continue sporadically into the present. An estimated 80% of all native Austrian crayfish populations disappeared in the 20th century (Pöckl, personal communication). A high percentage of these die-offs are associated with crayfish plague, which represents one of the major threats to the recovery of populations of native crayfish species in Central Europe [11]. For example, *Astacus astacus*, formerly a very abundant species in Europe, is now considered threatened by the International Union for Conservation of Nature and Natural Resources (IUCN) [12]. In many countries this economically valuable crayfish is on the Red List and its current harvest is probably less than 10% of the harvest rate before introduction of the crayfish-plague pathogen [13,14]. *A. astaci *was introduced from North America, where various species harbour the pathogen without showing clinical signs of infection. Crayfish-plague outbreaks among such populations often occur only under stress conditions. The introduction of resistant North American species like the signal crayfish (*Pacifastacus leniusculus*), the red-swamp crayfish (*Procambarus clarkii*) and the spiny-cheek crayfish (*Orconectes limosus*) http://www.issg.org/database has established a permanent reservoir for the pest in Europe. The transmission of the pathogen occurs via crayfish cadavers, crayfish-feeding fish [15], fish scales [16] and all kinds of equipment, which have been in contact with contaminated water [10]. The adaptive life style, high fecundity, and resistance to the pathogen make introduced crayfish species a potent bioinvador and the most dangerous vector for pathogen transmission.

Biflagellated secondary zoospores, measuring 8 × 12 μm, represent the infective unit of *A. astaci*. They target host tissue by various mechanisms including chemotaxis [17,18] on soft parts of the crayfish integument, especially at the joints, the bottom side of the abdomen and even near the eyestalks [19] as well as fresh wounds [20]. Once zoospores reach the upper lipoprotein-layer of the crayfish cuticle, they discard their flagellae, and develop a penetration peg, that weakens the lipid layer enzymatically [21]. Soon after the germ tube has penetrated the cuticle by mechanical force, the developing hyphae begin to secrete chitinases and proteases [22]. In this phase different chitinases [18] jointly degrade chitin polymers in order to release nutrients and facilitate further growth mainly parallel to the chitin fibrils of the endocuticula [23]. Based on their substrate affinity these enzymes can be classified into three groups: (i) endochitinases, which randomly cleave glycosidic linkages, generating free ends and long chitooligosaccharides that are processed by (ii) exochitinases (chitobiosidases), which release diacetylchitobiose (chitobiose) and (iii) N-acetylglucosaminidases (chitobiases), which hydrolyse chitobiose or release N-acetylglucosamine monomer from chitin chains [24,25].

High-level production of extracellular chitinase in the absence of substrate is one of the most prominent features of the specialised crayfish-parasite *A. astaci *[26,18]. The GH18 family-chitinase Chi1 was the first chitinase described for *A. astaci *[18]. Here we selected two additional members of this gene family as targets for an *A. astaci*-specific diagnostic assay. GH18 chitinases can be divided into three clusters, two of which (A and B) differentiated before the appearance of the eukaryotic lineage [27]. For example, fungal GH18 families comprise between one and twenty genes represented by members of all three clusters [28]. We demonstrate the temporally regulated expression of two novel members of the *A. astaci*-GH18 family. This functional constraint was regarded as a basic criterion for the development of a closed-tube diagnostic method for qualitative and quantitative detection of *A. astaci*. In conclusion, simultaneously targeting multiple chitinase sequences including the novel, functionally constrained chitinase sequences, facilitates a robust analysis of clinical samples with a maximum reduced chance of false-negative detection.

## Results

### Strain identification

Two putative *A. astaci *strains were recovered from healthy signal crayfish in two small streams in the Austrian province of Burgenland (Gb04 - Ganaubach and Z12 - Zöbernbach). A third strain (GKS07) was isolated from the subabdominal cuticle of a moribund noble crayfish specimen collected during an acute crayfish-plague outbreak in the lake „Gleinkersee” (Austrian province: Upper Austria) in March 2007 (Table 1). *ITS*-sequence data and constitutive chitinase secretion specific for *A. astaci *(Additional file 1) confirm the assumed species assignment for all three strains. The strain Gb04 was used to identify two new chitinase genes, test for their functional constraint and finally to develop the diagnostic assay for *A. astaci*.

The *Aphanomyces *strain LK29 was isolated from a healthy signal crayfish (*Pacifastacus leniusculus*). Physiological and genetic evidence showed that the strain does not fit into any previously identified group of *A. astaci*. It exhibited properties like repeated zoospore emergence and lack of sexual reproduction commonly associated with parasitic species. In contrast to *A. astaci*, the strain LK29 does not express chitinase constitutively during growth or sporulation. Phylogenetic analysis of *ITS *sequences (Additional file 1A) demonstrated clustering within the *A. laevis-repetans *clade [29]. In addition, a Blastn search with the *28SrDNA *sequence of LK29 (GenBank:GQ152606, this work) showed close homology to *A. laevis *(99%, GenBank:AF320584), but clear difference (97% identity) to the *A. astaci *strains Hö, FDL, GB04 and Z12 (AF320583, AF320582, GQ374534, GQ374535, respectively). Until their taxonomic status is fully elucidated the new isolate was assigned to *A. repetans*. This species is not capable of killing crayfish following standardised experimental infection and is characterised by a high growth rate, and germination in response to nutrients [30].

### Sequence determination of the novel A. astaci genes CHI2 and CHI3

Fungal species contain one to twenty GH18 chitinase family genes [28]. In order to develop a robust diagnostic assay for *A. astaci*, we asked whether the chitinolytic system of the pathogen would contain multiple genes of this ancient gene family widely expressed in archea, prokaryotes and eukaryotes [31].

**Figure 1:**
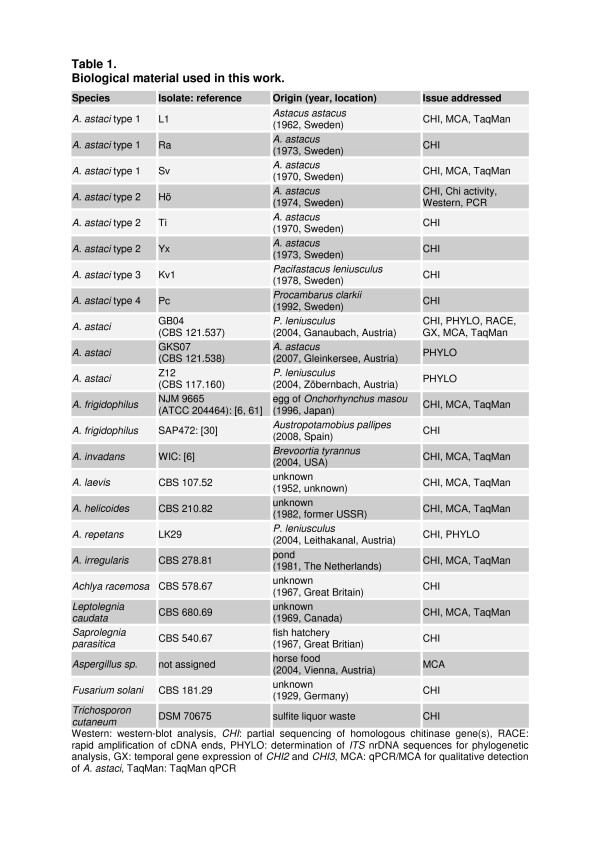
**Biological material used in this work**.

**Figure 2:**
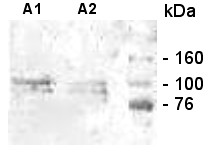
**Western-blot analysis of chitinfree PG1-supernatant of a ten-day old *A. astaci *(strain Hö) broth culture**. Two bands of about 100 kDa and slightly below this size were detected by antibodies A1 and A2 raised against epitopes in the catalytic domain of the first *A. astaci *GH18 chitinase family member Chi1.

**Figure 3:**
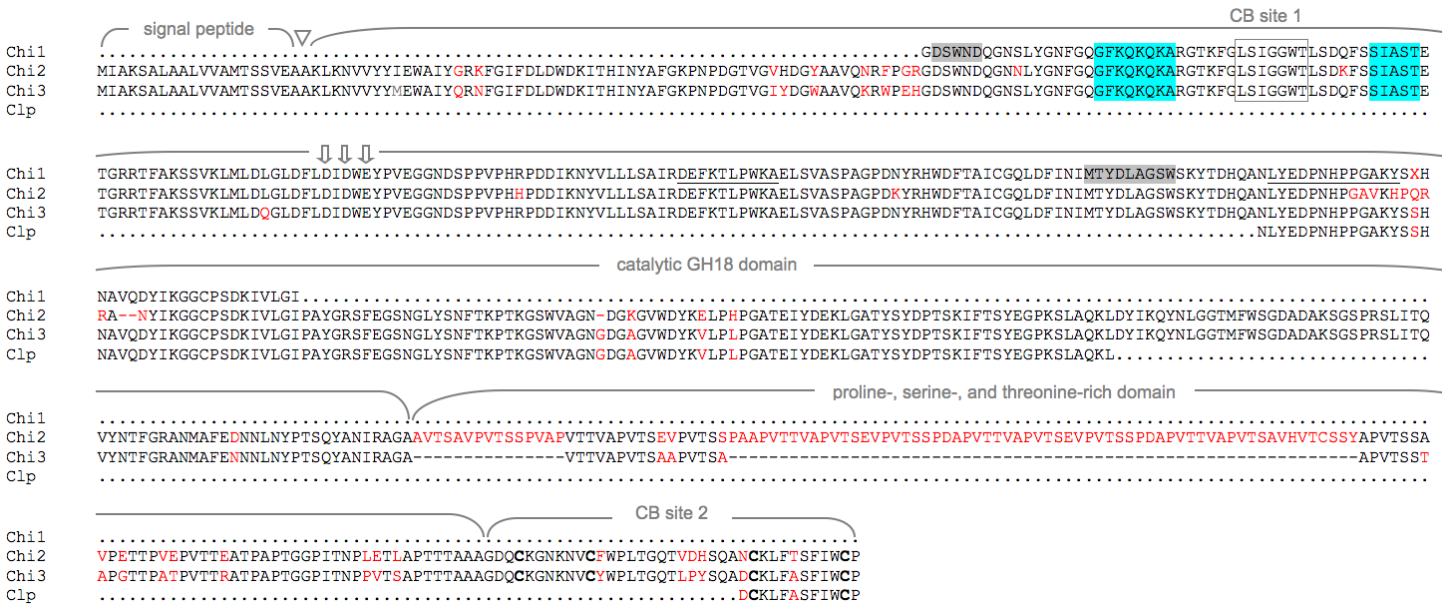
**Domains completeley homologous in the novel chitinases *Chi2 *and *Chi3 *as well as in the first *A. astaci *chitinase (*Chi1*, GenBank**:AJ416354, [18]) **were selected as primer target sites in the diagnostic assays for *A. astaci***. In blue: primer target sites. Note that only the homologous part of Chi1 is shown. The chitinase-like protein Clp mRNA (GenBank:FJ439176) was amplified from cDNA, but failed to amplify from genomic DNA for unknown reasons (data not shown). Chi1 peptide sequences selected to generate antibodies for Western blot analysis are underlined. Highly conserved motifs in the GH18 domain (grey boxes) were selected as primer target sites to identify the homologous genes of related oomycetes and relevant fungi (see text). Dots indicate missing sequence homology. The triangle marks the signal peptide cleavage site in Chi2 and Chi3. The catalytic-site residues D154, D156 and E158 putatively required for catalytic activity [27] are indicated by vertical arrows. Residues given as red or black letters represent mismatches and conservative changes, respectively. The conserved cysteines in the CB site 2 are highlighted in bold.

As indicated by the two cross-reacting bands detected in western-blot analysis with antibodies raised against the catalytic GH18 domain, *A. astaci *contains more than one chitinase-like protein (Figure 2). Therefore, we attempted to identify homologous genes using PCR amplification with consensus primers targeting the amino acid motifs DSWND and AGSW (Figure 3). For various *A. astaci *strains representing all four genotype groups described (type A: L1, Sv, Ra; B: Hö, Yx, Ti; C: Kv; D: Pc; [32]) and the Austrian strain Gb04 isolated in this work (Figure 1), partial GH18 domain sequences were amplified and subsequently sequenced. Analysis revealed a mixture of sequences derived from two new chitinase genes (*CHI2 *and *CHI3*, see below), as concluded by retrospective evaluation. Only synonymous substitutions were found in these genes (data not shown). Starting from the consensus sequence obtained for the "core" of *CHI2 *and *CHI3 *mRNAs, their complete mRNA sequences were identified by Rapid Amplification of cDNA Ends (RACE)-PCR and submitted to the GenBank (accessions FJ439177 and FJ386997, respectively).

Genomic DNA amplified with gene specific primers designed near the start and stop codons of *CHI2 *and *CHI3*, yielded fragments of 1810 bp and 1617 bp for *CHI2 *and *CHI3*, respectively. Subsequent sequence analysis performed with a primer-walking strategy (data not shown) confirmed the absence of the consensus sequence for exon-intron junctions (5'-GTRNGT...YAG-3' [33]) and identity of cDNA and genomic sequences (GenBank:DQ974157 and FJ457089 for genomic sequences of *CHI2 *and *CHI3*).

### Characterization of cDNA and deduced amino acid sequences of CHI2 and CHI3

Without the poly(A) tail, the cDNA sequences of *CHI2 *and *CHI3 *measure 1807 and 1591-bp in length and exhibit a relatively high guanine and cytosine base content of 59.9% and 60.3%, respectively, a typical feature of oomycete genes [34]. *CHI2 *and *CHI3 *code for open reading frames of 596 and 522 amino acids (Figure 3) with molecular masses of 64.0 kDa and 56.7 kDa and isoelectric points of pH 6.14 and 6.63 predicted for the mature secreted enzymes Chi2 and Chi3 (see below), respectively. The mRNAs possess an identical 42-bp 5' untranslated region (UTR) carrying the major part of the oomycete consensus sequence for the start site of transcription (TATTCAATTTGCCAT, [33]). The 3' UTRs of *CHI2 *and *CHI3 *contain the polyadenylation signal WAUAAC (W = A or T) [35] (Additional file 2). In both genes the translation start codon is part of the eukaryotic consensus ACCATGA [33]. The enzymes are predicted to be cleaved by signal peptidase between positions A20 and A21 producing a hydrophobic signal peptide of 20 amino acids (Figure 3).

**Figure 4:**
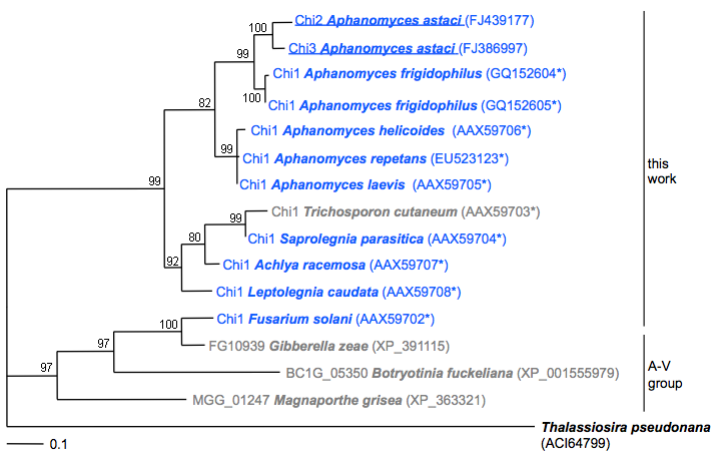
**The *A. astaci *chitinases Chi2 and Chi3 possess an oomycete type-GH18 catalytic domain**. Maximum likelihood phylogenetic analysis was performed with TreePuzzle using the diatom *Thalassiosira pseudonana *as an outgroup. Oomycete and fungal sequences are given in blue and grey, respectively. GenBank accession numbers of partial or complete amino acid GH18 domain sequences are indicated in parentheses. The scale bar represents 0.1 substitutions per site. The numbers at the nodes are quartet puzzling values indicating the frequencies of occurrence for 1,000 replicate trees and can be interpreted in much the same way as bootstrap values. The group A-V - one of six separate fungal groups classified [27,28] - showing the closest homology to the sequences identified in this work, is represented by two members. An asterisk denotes partial sequences.

Overall, the deduced amino acid sequences of *CHI2 *and *CHI3 *are highly homologous with an identity of up to 79.0% (overlapping residues 1 to 596 and 1 to 522, respectively). The proline-, serine-, and threonine-rich domain [36] of Chi2 contains extra residues resulting in an extended amino acid sequence of the whole protein compared to Chi3 (Figure 3). This domain also represents the most heterologous part of the enzymes regarding primary sequence. Chi2 and Chi3 possess an oomycete-type catalytic GH18 domain (A21 to G400/403, Figure 4). It contains a conserved chitin-binding (CB) site [37] (CB site 1 in Figure 3), and an active site consensus [LIVMFY] - [DN] - G - [LIVMF] - [DN] - [LIVMF] - [DN] - x - E (Prosite no. PS01095) being variant at one position (Additional file 3). The catalytic-site residues D154, D156 and E158 are putatively required for catalytic activity [27]. A second putative, highly homologous CB site was identified in the C-terminal part of the chitinases (CB site 2 in Figure 4). It contains four cysteines, instead of the five residues found in a diatom chitinase (GenBank:EED92972) or six in most insect chitinases [38].

Chi2 and Chi3 sequences contain sites for amidation, N-myristoylation or O-linked glycosylation (Additional file 4). The latter type of glycosylation predicted for the C-terminal protein parts occurs often at serine and threonine residues that would otherwise be phosphorylated; one illustration of the complex interplay among eukaryotic post-translational modification systems [39]. N-glycosylation at N165/165 (site: NDS) and N296/298 (site: NFT) was predicted for Chi2/Chi3, respectively. These posttranslational modifications may account for the discrepant masses deduced from primary protein sequences and calculated on the basis of the electrophoretic mobility (Figure 2). Putative sites for C-linked glycosylation (C-mannosylation, [39]) were not found. The tripeptide 'RGD' mediating cell adhesion (R81 to D83) was predicted for Chi2. Potential sites for phosphorylation at serine, threonine and tyrosine residues are listed in the Additional file 4.

### Temporal mRNA expression analysis for CHI2 and CHI3

Next, we verified that target genes selected for the DNA-based diagnostic crayfish-plague assay are subject to functional constraint. This could be assumed if temporal expression of target genes significantly changes during physiological conditions relevant to the infection *in vivo*.

The *CHI2 *and *CHI3 *mRNA copy numbers expressed in the *A. astaci *mycelium, grown in chitin-free culture were quantified over three days at intervals of twelve hours using one-step qRT-PCR. A partial sequence of the nuclear gene *NDUFV1 *encoding the mitochondrial protein NADH dehydrogenase (ubiquinone) flavoprotein 1, which is part of mitochondrial respiratory chain complex I, was identified in this work (data not shown, GenBank:EU500726). We used this sequence as target for an endogenous positive control qRT-PCR assay reporting deviations in extraction, reverse transcription and PCR amplification including mRNA integrity, quality, and quantity. Overall, levels of *NDUFV1 *mRNA changed only slightly across the time points studied (< 2.5-fold), including, however, expression changes which were near or below the level of significance (p = 0.05) but not matching the temporal expression patterns of the chitinases. In detail, the dynamic growth of the mycelium during the first hours in drop culture (12 to 24 hours, [18]) was reflected by the higher *NDUFV1 *expression found after 12 and 24 hours of culture (P = 0.03 and 0.07, respectively). Mycelium growth reached its plateau after 72 hours of incubation. The decreasing energy requirement and the beginning of autolytic processes at this stage are reflected by a lower *NDUFV1*-transcript copy number (P = 0.05 for expressions at 72 and 24 hours).

**Figure 5:**
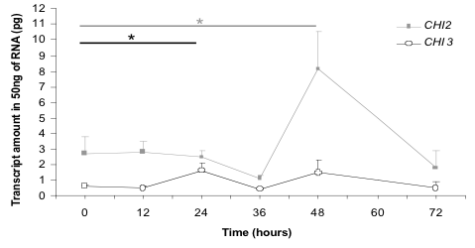
**Significant changes of temporal expression of *CHI2 *and *CHI3 *mRNAs**. The transcript abundance changes during 72 hours of growth in chitinless, liquid PG-1 medium. The significant differences in temporal expression indicate functional constraint and are in accordance with the plurifunctionality of GH18 family members, respectively. Error bars (only the positive error bar is shown) represent the standard errors of the mean obtained from three independent time-course experiments. The asterisk designates significance at p < 0.05.

The chitinase genes *CHI2 *and *CHI3 *were both constitutively expressed in mycelium grown in a medium lacking the substrate chitin. However, different mRNA amounts and temporal expression patterns, including the time point at which the maximum level was reached, were observed (Figure 5). Most prominent was the significant maximum in the *CHI2 *mRNA level reached after 48 hours (P = 0.013).

Analogous to data obtained for *CHI1 *[18], we demonstrated, exemplarily for *CHI3*, that neither the amplitude of expression nor its pattern was influenced by substrate addition (0.6% colloidal chitin instead of glucose, data not shown).

### Assay development for qualitative and quantitative detection of A. astaci in clinical samples based on chitinase gene sequences

Compared to other crayfish-afflicting oomycetes, permanent chitinase expression and activity represents a unique feature of *A. astaci *[18,40]. Due to the assumed functional constraints demonstrated by the significant alterations of temporal gene expression (Figure 5), its chitinolytic system was chosen as a target for the development of a diagnostic test.

#### qPCR/MCA

**Figure 6:**
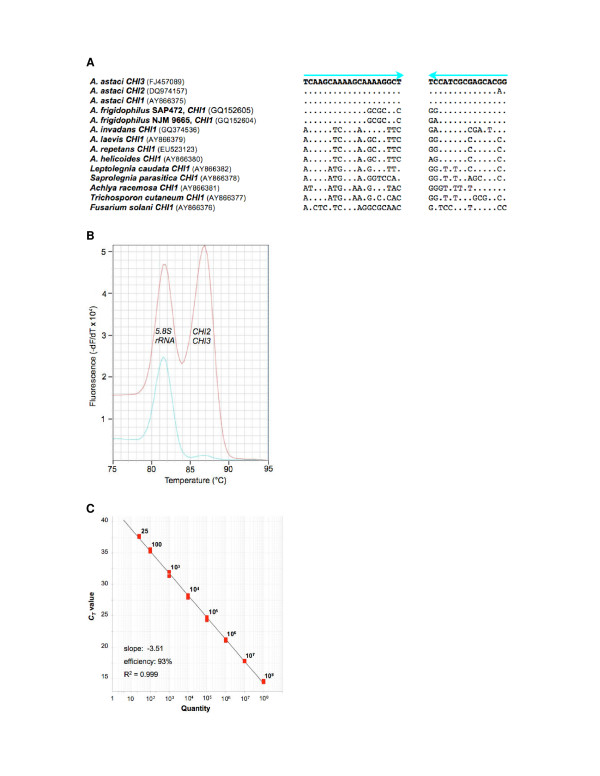
**Qualitative and quantitative detection of the oomycete *A. astaci***. **A: **Diagnostic qPCR/MCA primers (blue arrows) target *A. astaci*-specific sites in the homologous chitinase genes *CHI1*, *CHI2 *and *CHI3*, but not homologous sequences of related oomycetes and fungi. Parentheses contain GenBank accession numbers. Dots and letters represent identical and substituted nucleotides compared to the *A. astaci *sequence, respectively. **B: **Qualititative detection of *A. astaci *by qPCR/MCA. The left and right peaks are derived from amplification of the endogenous control, and the chitinase genes *CHI2 *&*CHI3*, respectively. Red plot: *A. astaci*, blue plot: *A. frigidophilus*. **C: **Quantitative detection of *A. astaci *by TaqMan qPCR. The standard curve of the assay demonstrates quantification down to 25 copies.

A BLASTp search with the deduced amino acid sequence of *CHI1 *as query identified two conserved motifs within the GH18 chitinase domain (83-DSWND and 229-MTYDLAGSW, Figure 3). The nucleotide sequences of these motifs were used as target sites for the design of degenerated PCR primers. Using these primers we were able to amplify and sequence the homologous sequences of nine strains from eight oomycete species and two fungi which are known to live on or in proximity of crayfish (species and GenBank accessions in Figure 6a). On the basis of these sequences, we designed a diagnostic primer pair producing a 93 bp-amplicon from each of the three related chitinase genes (*CHI1*: [18], *CHI2 *and *CHI3: *this work, Figure 6a). Its melting temperature of 86.7°C in MCA was regarded as criterion for identification of an *A. astaci *strain. For assay robustness the chitinase primer pair was multiplexed with primers targeting the *5.8S rRNA *gene as an endogenous control (Additional file 5) and yielding a peak in MCA at 81.5 to 83.5°C depending from the species investigated.

The qPCR/MCA assay was tested for specificity against the oomycetes *A. frigidophilus*, *A. invadans*, *A. laevis*, *A. helicoides*, *A. irregularis*, and *Leptolegnia caudata*. Only the endogenous control was recorded, but not the *A. astaci*-specific chitinase peak.

qPCR/MCA-based detection of *A. astaci *was used to elucidate several spontanous crayfish mortalities in Austrian waterbodies. In detail, *A. astaci *was identified as causative agent of acute crayfish-plague outbreaks among noble crayfish inhabiting a small unnamed pond-system (Hartberg, district Hartberg, province Styria), in the noble crayfish-pond Bäckerteich (Velden am Wörthersee, district Villach-Land, province Carinthia), in the brook Hahntrattenbach near St. Andrä (district Wolfsberg, province Carinthia) known for its large stone crayfish population and in a noble crayfish population of the lake Gleinkersee (Roßleithen, district Kirchdorf an der Krems, province Upper Austria). *A. astaci *was also detected by MCA in necrobiopsy pools each derived from up to five euthanised signal crayfish specimens collected at the streams Ganaubach, Zöbernbach, Strem, Tauchenbach and Güns (province Burgenland). Clinical samples tested positive by MCA were subjected to pathogen isolation. In case of isolation failure the qPCR/MCA amplicon was sequenced.

#### TaqMan qPCR

For sensitive detection of the pathogen, but also for quantification of agent levels in susceptible crayfish and carrier crayfish, a TaqMan-probe-based qPCR assay was developed. TaqMan qPCR uses the same primers as qPCR/MCA except the additional nucleotide at the very 5' end of the reverse primer compared to qPCR/MCA. Using amplicon standards with known copy numbers spiked into genomic crayfish DNA, a quantitative detection limit of 25 target sequences was determined (Figure 6c). No amplication, *i.e*. *C*_*T *_> 50, was obtained for *A. frigidophilus*, A. *invadans*, *A. leaevis *and *A. irregularis*, In the case of the oomycete species *A. helicoides *and *Leptolegnia caudata *a cross-amplification signal corresponding to 28 and 44 copies was detected, respectively.

## Discussion

Qualitative detection of two or multiple target sequences by MCA has been reported before. Single-tube SNP genotyping [41], sex determination [42], identification of methylation in promoter sequences [43] or the simultaneous detection of multiple pathogens [44,45] are exemplarily mentioned.

In this work we have used MCA of multiplex qPCR [46] for rapid species identification of the crayfish-plague pathogen *A. astaci *in a closed-tube format. The diagnostic assay for qualitative detection is highly discriminative, robust, inexpensive, and reliable.

High discrimination was aimed at since new *Aphanomyces ITS *sequences, probably representing new *Aphanomyces *spp. and including sequences closely related to *A. astaci *were reported [47,48]. Current molecular techniques for *A. astaci *detection based on *ITS *sequences suffer from a lack of specificity ([47,48], Additional file 6), or are laborious and time-consuming due to agarose electrophoresis and subsequent amplicon sequence analysis [11]. To facilitate unambiguous species identification, we considered the unique feature of constitutive chitinase gene expression of *A. astaci*, not found in closely related *Aphanomyces *species [18,26]. In a search for additional GH18 family members the novel chitinase genes *CHI2 *and *CHI3 *were identified in this work. The genes differ in their 3' UTRs including variant putative polyadenylation signals. Their temporal mRNA expressions change differently during mycelium growth in chitin-free medium. The deduced extracellular protein sequences are different in proline-, serine-, and threonine-rich domain size, and either possess or lack a putative cell attachement site. This speaks in favour of a joint action during the infection process. Therefore, we regarded *CHI2 *and *CHI3 *as different members of the GH18 gene family rather than allelic sequences. Altogether, three genes (*CHI1*, *CHI2 *and *CHI3*) encoding constitutively expressed GH18 chitinases in the absence of chitin were identified as unique characteristics of *A. astaci *and selected as targets for species-specific detection.

Assay robustness, characterised by a low risk of false negatives related to genotypic variation of pathogenic strains, was another issue for assay design. This was especially important since *A. astaci *belongs to the group of asexual organisms, for which a low level of genetic variation turns out to be the exception rather than the rule [49]. We argued that targeting one or even several functionally constrained sequences would restrict the genotypic variations allowed. The novel chitinase genes *CHI2 *and *CHI3 *being functionally constrained as concluded from their significant changes in temporal mRNA expression during growth (Figure 5) were regarded to be appropriate candidates to achieve this aim. Together with the first member of the GH18 gene family of *A. astaci *(*CHI1*: [18]) they served as targets in the diagnostic assays based on qPCR/MCA or TaqMan qPCR. In the qPCR/MCA-based assay for qualitative detection, a further level of robustness was achieved by multiplexing with a primer pair targeting the *5.8S rRNA *gene as an endogenous control. This DNA sequence is naturally present at multiple copies [50] and harbours two completely homologous primer target sites in each experimental oomycete species (Figure 6a). The simultaneous amplification of this *5.8S rRNA *sequence controling for the DNA extraction and amplification steps reduces the chance of false negative detection due to insufficient sample quality. The chitinase gene targets and the endogenous control can be considered to be present at comparable copy numbers [50,28]. Therefore, if non-limited primer concentrations are applied like here, the simultaneous amplification of more than one target in a single PCR, *i.e*. multiplexing, leads to competition between multiple targets for a finite number of reagents. Representing a welcomed side effect, this further enhances assay discrimination (see above). Co-amplification of an endogenous control adds another level to assay robustness and represents an improvement compared to the *ITS1*-based TaqMan minor-groove binder qPCR assay for *A. astaci*-detection reported recently [51]. Coextraction of an homologous (competitive) internal positive control (IPC) with the clinical samples and coamplification in the qPCR or qPCR/MCA assays with the same primers used for the target DNA ensures accurate control of the entire molecular assay and represents the state of the art for internal controls. It was shown that the addition of an IPC at levels resulting in 100 copies per PCR did not affect the amplification of the target sequence [52,53]. A competitive IPC compatible with the qPCR/MCA and TaqMan qPCR assays developed in this work is presented as Additional file 7.

Another level of diagnostic uncertainty in the assay developed for *A. astaci *detection [51] is added by the use of a synthetic amplicon mimicking one of the closest relatives, *A. frigidophilus*. This approach supposes the intragenomic homogeneity of the ITS regions which has already been rebutted in many organisms [54,55]. The addition of a minor-groove binder to a TaqMan probe in the assay reported by Vralstad *et al*. allows to use shorter probes. However, probe cost increases by about 2.5-fold compared to our conventional TaqMan qPCR designed for quantitative detection. It also elevates the chance of detection failure when varying genotypes are present. Generally, the avoidance of false negatives represents a major challenge in molecular diagnostics. Particularly, in TaqMan qPCR assays the possibility of false-negative testing poses a substantial problem because mutations within the probe-binding site can prevent annealing of the probe and subsequent detection [56,57]. For example, TaqMan qPCR failed to detect any target with more than two mutations at the probe-binding site in contrast to a dye-based assay [56]. The dilemma of false-negative detection due to probe-binding site variation can be overcome, for example, by combining a DNA probe with a fluorescent, double-stranded DNA-binding dye for specific nucleic acid quantification by probe-based qPCR and MCA [58]. In this case the dye would report a detection failure if the probe-binding site of a clinical specimen is mutated. However, "compensation" for mutations in the probe-binding site is no longer an issue if only two instead of three regions of conserved sequence are required for assay design as in the dye-based qPCR/MCA developed in this work. If very limited prior target sequence information exists from a population of interest like in our case, a dye-based detection approach represents a favourable strategy for species confirmation.

A welcomed side effect of dye- instead of probe-mediated monitoring is the cost reduction for screening clinical samples.

Last but not least, the reliability of the diagnostic assay was proven on a set of relevant related pathogens and during an acute crayfish-plague outbreak in the small, noble crayfish (*Astacus astacus*) population inhabiting the lake "Gleinkersee" located at an altitude of about 800 meters above sea level at the foothills of the Austrian Alps. In addition to qPCR/MCA typing (not shown), the presence of the pathogen *A. astaci *was independently confirmed by *ITS*-sequence analysis and testing for constitutive chitinase activity (*A. astaci*-strain GKS07 in Additional file 1). Finally, the *A. astaci *strain GKS07 was isolated on PG-1 agar from an infected noble crayfish. Numerous crayfish individuals were found to be affected but were still alive during the outbreak of late March 2007. At that time the ice of the lake Gleinkersee was melting and the physiological activities of both pathogen and victim would have been expected to be at a minimum. These circumstances strongly indicated the acuteness of the outbreak. The suspicion of a deliberate introduction of the pathogen could not be confirmed by an inquiry led by the local criminal investigation department. Fish stocking performed in autumn 2006 may be the most likely source of disease transmission.

Sensitive quantitative detection of the crayfish-plague pathogen is currently of increasing importance for screening natural non-native crayfish populations or for certifying a pathogen-free status of hatchery fish before introduction into natural habitats or aquaculture facilities. Samples of fish transport water including sediments can be filtered via membrane filters [59] and subsequently screened by TaqMan qPCR (see Results and Additional file 8). This circumvents pathogen transmission via transport water, fish faeces, mucus and scales.

## Conclusion

The identification of two new chitinase genes showing specific patterns of constitutive temporal expression in the absence of substrate has facilitated the development of a discriminative, robust and reliable method for qualitative and quantitative detection for *A. astaci*.

## Methods

### Biological material

Isolates of *Oomycetes *and related fungi used to validate the molecular assays were either obtained from The Centraalbureau voor Schimmelcultures (CBS) Fungal Biodiversity Centre (Utrecht, The Netherlands), the German Collection of Microorganisms and Cell Cultures (DSMZ) (Braunschweig, Germany), the American Type Culture Collection (ATCC) or cultured from lesioned tissue by standard methods [60,61]. The *A. astaci*-types 1 to 4 were purchased from Lage Cerenius (Uppsala University, Uppsala, Sweden). Javier Diéguez-Uribeondo (Real Jardín Botánico CSIC, Madrid, Spain) provided the *A. frigidophilus *isolate SAP472 [29]. A DNA aliquot of *A. frigidophilus *NJM 9665 [6,62] and *A. invadans *WIC [6] was obtained from Mark W. Vandersea (Center for Coastal Fisheries and Habitat Research, National Ocean Service, National Oceanic Atmospheric Administration, Beaufort, North Carolina, USA).

The Austrian *A. astaci *strains Gb04, Z12, and the *A. repetans *strain Lk29 were isolated from dissected melanised spots found in the integument of signal crayfish [19]. The *A. astaci *strain GKS07 was grown out of a moribund noble crayfish collected during an acute crayfish-plague outbreak. Melanised necrobiopsies were incubated in peptone-glucose (PG1) medium (3 g/l glucose, 6 g/l peptone, 0.37 g/l KCl, 0.17 g/l MgCl_2_·6H_2_O, 0.15 g/l CaCl_2_·2H_2_O, 20 mg/l FeCl_3_·6H_2_O, 44 mg/l Na_2_EDTA, 13 mM sodium phosphate buffer (pH 6.3); [63]) for three days at 18°C [19] in a humidified chamber and subcultured every two weeks on PG1 agar medium. The same growth and subculturing conditions were applied to the strains obtained from the culture collections.

Fungal contamination of oomycete culture encountered when culturing the *A. astaci *strain Z12 and the *A. repetans *strain LK29 were overcome as follows. A piece of agar culture was incubated for one day at 20°C in autoclaved pond water (pH 6.5 to 7) collected at the central biotop of the University campus. This depletion of nutrients induced the sporulation of the oomycete [64]. Under an inverted microscope the swimm spores were aspired into a 100 μL Gilson pipette and re-cultured on PG1 agar medium.

A fungus isolated from horse food was assigned to *Aspergillus sp*. based on morphological evaluation and added to the strain collection of the Institute of Bacteriology, Mycology and Hygiene (University of Veterinary Medicine, Vienna).

An overview on the biological material used in this work is presented in Table 1.

### Species assignment of Austrian Aphanomyces strains

ITS sequences of nuclear rDNA were analysed to allow species assignation of the Austrian *A. astaci *strains GB04, GKS07, and Z12 as well as of the *A. repetans *strain LK29 (Table 1, Additional file 1). For this purpose DNA was extracted from 25 mg drop culture mycelium using the DNeasy Tissue Kit (Qiagen, Hilden, Germany). A DNA fragment of about 1,000 bp was amplified and sequenced using the universal primers V9D (5'-TTACGTCCCTGCCCTTTGTA) [65] and LSU266 (5'-GCATTCCCAAACAACTCGACTC, [66]). Sequences obtained were compared with reference homologs of *Aphanomyces *[29] retrieved from GenBank. For sequence alignment the CodonCode Aligner software (version 3.0.1; CodonCode, Dedham, USA) was used. Molecular phylogenetic relationships were reconstructed using default settings in a program package for quartet-based maximum-likelihood analysis (TREE-PUZZLE, version 5.2 [67]) and TreeView for graphical illustration [68].

Additional evidence for species assignation was obtained from sequence analysis of the large subunit ribosomal RNA gene using the primers nuLSU-5' (5'-CGCTGATTTTTCCAAGCCC) and nuLSU-3' (5'-GAGATAGGGAGGAAGCCATGG) for amplification and sequencing.

Thus far *A. astaci *represents the only species within the genus *Aphanomyces *known to produce significant amounts of chitinase in chitin-free medium [18]. This unique feature was additionally used for species assignment. In detail, chitinase activity accumulated in broth culture supernatant was measured in a reaction volume of 100 μL containing 5 mM sodium-phosphate buffer (pH 7), 180 μM 4-methylumbelliferyl-β-D-N,N',N''-triacetylchitotrioside (4-MU-chitotrioside; Sigma-Aldrich, Vienna, Austria) as substrate, and 75 μl of supernatant [18]. Following incubation at room temperature for 10 min, the fluorescence intensity was evaluated under UV light.

### DNA isolation from mycelium of oomycetes

The mycelium was transferred to a 2 ml-extraction tube containing 0.7 g Precellys^® ^ceramic beads of 1.4 mm diameter (Peqlab Biotechnology, Erlangen, Germany) and 180 μl buffer ATL, the lysis buffer of the DNeasy^® ^Blood & Tissue Kit (Qiagen, Hilden, Germany). Samples were homogenised twice for 15 s at 5000 rpm using the MagNA Lyser (Roche). Further isolation was performed according to the protocol "Purification of Total DNA from Animal Tissues (Spin-Column Protocol)" provided by the manufacturer.

### De novo sequencing of partial GH domain using degenerate PCR primers

Partial GH18 domains of chitinases from various *A. astaci *strains representing all four genotype groups described (A: L1, Sv, Ra; B: Hö, Yx, Ti; C: Kv; D: Pc; [32]), the Austrian strain Gb04 isolated in this work and six related oomycete species (*A. laevis*, *A. helicoides*, *A. repetans*, *A. irregularis*, *Saprolegnia parasitica*, *Achlya racemosa*, *Leptolegnia caudata *(Table 1) were amplified using the primers SEQ685F (5'-CCGGAGACTCGTGGAACGAC) and SEQ1159R (5'-TTGCTCCAGCTGCCCGC). Primers targeting the amino acid motifs DSWND and AGSW, respectively, amplified an approximately 475-bp product by qPCR. The 20-μL reaction consisted of 0.4 × EvaGreen™ dye (Biotium, Hayward, USA), 4 mM MgCl_2_, 200 μM of each dNTP, 375 nM of each primer, 2 μl template DNA, 1 U GoTaq^® ^DNA polymerase - a proprietary formulation of *Taq *DNA polymerase (Promega, Madison, USA), and 1 × Colorless GoTaq^® ^Flexi Reaction Buffer (Promega) not containing magnesium. Amplification was performed in the Rotor-Gene 6000 (Corbett Life Science, Sydney, Australia) using denaturation for 4 min at 94°C, amplification for 35 cycles (1 min at 94°C, 1 min at 63°C and 1 min at 72°C), and final elongation of 7 min at 72°C followed by MCA.

Amplicons from *Fusarium solani *and *Trichosporon cutaneum*, representing fungi, were obtained with the degenerate primer SEQuni-F (5'-CGCCGGAGAYTCTTGGAAYGA, Y = C or T) in combination with the primer SEQuni-R (5'-CCAGCATAGTCGTAGGCCAT) targeting the amino acid motifs xxDSWND and MTDYAG, respectively.

Agarose gel electrophoresis was used to the determine amplicon size. The MSB^® ^Spin PCRapace Kit (Invitek, Berlin, Germany) was used for amplicon purification in case of a single band showing the expected length. Multiple bands were excised from the gel and purified with the Xact DNA Cleanup kit (genXpress, Wiener Neudorf, Austria).

The BigDye^® ^Terminator sequencing chemistry (Applied Biosystems, Foster City, USA) was used for sequence analysis of amplicons performed at VBC Genomics Bioscience Research GmbH (Vienna, Austria).

### Identification and phylogenetic analysis of GH18 domains

The GH18 domain in the amino acid sequences of *CHI2 *and *CHI3 *were identified using the Reversed Position Specific Blast (rpsblast) search modus and the conserved domain database [69]. Domain sequences were aligned to GH18 domain sequences of related species with the ClustalW alignment program implemented in the graphical multiple sequence alignment editor SeaView version 4 [70]. Quartet-based maximum likelihood analysis for aligned amino acid sequences was performed using TreePuzzle with default settings [67]. The graphical display of the phylogram was generated as described above.

### Western blot analysis of A. astaci culture supernatant

The peptides DEFKTLPWKAE and LYEDPNHPPGAKY were selected from the deduced amino acid sequence of the *A. astaci *gene *CHI1 *(GenBank:AJ416354). Conjugates of these peptides with bovine serum albumin (BSA) were obtained from PSL GmbH (Heidelberg, Germany). Coupling to BSA was achieved via the SH group of a cysteine residue introduced at the C terminus of the peptide to be synthesised. Conjugates were used for the production of polyclonal rabbit serum antibodies served as primary antibodies. Peroxidase-labelled goat anti-rabbit IgG antibodies (K&P Laboratories, Gaithersburg, USA) were used as secondary antibodies.

Western-immunoblot analysis was performed as follows. The *A. astaci *strain Hö was grown in broth culure. The culture supernatant was boiled for 5 min in a buffer consisting of 25 mM Tris-HCl (pH 6.8), 2.2% sodium dodecyl sulfate (SDS), 15% glycerol and 0.001% bromophenol blue. Insoluble debris was removed by centrifugation. Proteins were resolved by SDS-polyacrylamide gel electrophoresis on a 12% polyacrylamide Tris-glycine gel and electroblotted onto a polyvinylidene difluoride (PVDF) membrane (Bio-Rad Laboratories, Hercules, USA) using a tank blot system (Bio-Rad). The Opti-4CN™ substrate detection kit (Bio-Rad) was used for colorimetric detection of secondary antibodies conjugated to horseradish peroxidase.

### Determination of complete cDNA- and genomic-DNA sequences for CHI2 and CHI3

Mycelium derived from the *A. astaci*-strain Gb04 was grown in liquid PG1 medium for three days and transferred to fresh medium for another 24 h. Total RNA was isolated from mycelium using the Plant and Fungi Protocol provided with the RNeasy Plant Mini Kit (Qiagen). Treatment with DNase I (Promega, Mannheim, Germany) was performed at 37°C for 40 min according to the supplier's instructions. The complete cDNA sequences of *CHI2 *and *CHI3 *were generated by RACE-PCR using the 5'/3' RACE Kit (Roche Applied Science, Vienna, Austria).

To amplify genomic sequences corresponding to the cDNAs determined, we designed primers in the region of the start and stop codons of *CHI2 *and *CHI3*. The common forward primer (Chi5'f: 5'-AGCAAACTGCAACAAGCATG) targeting a region immediately upstream of the start codon of putative *CHI2 *and *CHI3 *genomic sequences, was combined with a gene-specific reverse primer binding adjacent to the stop codon (Chi2.3'r: 5'-GGGCACCAGATGAACGACGC or Chi3.3'r: 5'-ACTAACATACACAACGAATGCGC for *CHI2 *and *CHI3*, respectively). The matching fragment size between cDNA and respective DNA sequences shown by agarose gel electrophoresis, and the identity of genomic and cDNA sequences identified by a primer-walking strategy (data not shown), were considered as experimental demonstration for the absence of intronic sequences within *CHI2 *and *CHI3 *genes.

### In silico analysis of amino acid sequences deduced from CHI2 and CHI3

Multiple matching subsegments in two protein sequences were identified with the LALIGN program http://www.ch.embnet.org/software/LALIGN_form.html implementing the algorithm of Huang & Miller [71].

The theoretical isoelectric points for the protein sequences were calculated using the Protein Isoelectric Point menu within the Sequence Manipulation Suite [72].

The presence and location of signal peptide cleavage sites in the amino acid sequences of *CHI2 *and *CHI3 *were predicted with the SignalP 3.0 Server http://www.cbs.dtu.dk/services/SignalP;[73]). Protein phosphorylation at serine, threonine or tyrosine residues was predicted with the NetPhos 2.0 Server [74]. Putative sites for amidation, N-myristoylation and cell attachment were identified by a protein pattern search against the Prosite database http://www.expasy.org/prosite/; [75]). O-, N-, and C-glycosylated sites were predicted with EnsembleGly - a web server for prediction of O-, N-, and C-linked glycosylation sites with ensemble learning [39].

### Transcript quantification by real-time reverse transcription PCR (qRT-PCR)

Propagules of the strain Gb04 were grown in PG1 medium for three days, washed in fresh medium for 2 min and transferred to another portion of fresh medium (time point 0). Twelve, 24, 36, 48 or 72 hours later the mycelium was shortly washed with distilled water, quick-frozen in liquid nitrogen and stored at -80°C. RNA was isolated from three independent samples grown per time point.

For quantification of transcript mass expressed from the chitinase genes *CHI2 *and *CHI3 *as well as the endogenous positive control *NDUFV1*, sense strand transcript standards were generated by *in vitro *transcription from a PCR product template tailed with the T7 phage promoter sequence. In more detail, for template construction a minimum sequence of 19 bases (5'-TAATACGACTCACTATAGG) required for efficient transcription was selected out of the 23 nt T7 phage promoter sequence and added to the 5' end of the respective PCR primer. *In vitro *transcription was performed with the RNAMaxx™ High Yield Transcription Kit (Stratagene, Amsterdam, The Netherlands) according to the manufacturer's instructions. Transcription was terminated by adding 1 μl DNase I (10 units/μl RQ1 RNase-Free DNase, Promega) and incubation at 37°C for 40 minutes. The amount of the *in vitro *transcript was determined by UV-absorbance measurement performed at 260 nm on a GeneQuantII RNA/DNA Calculator (Pharmacia Biotech, Cambridge, UK). Ten-fold serial dilutions were used as absolute concentration standards.

The 10-μl one-step qRT-PCR contained 125 nM of each primer (5'-CCATCACGAACCCCCTTGAG and 5'-GGGCACCAGATGAACGACG for *CHI2*, 5'-GTGGCCCCATCACGAACC and 5'-ACTAACATACACAACGAATGCGC for *CHI3*, 5'-TCGGCTGTCGCACTTCTACA and 5'-ATCCACCCCGTTCCTTCG for *NDUV1*), 75 nM TaqMan probe (Hexachloro-6-carboxyfluorescein (HEX)-5'-CTGCGGCCAATGTACCCCTTGCC black-hole quencher 1 (BHQ1) and 6-carboxyfluorescein (FAM)-5'-TTGTTGCCCTTGCACTGGTCGCC-BHQ1 for *NDUV1 *and *CHI2*/*CHI3*, respectively), 0.1 μl of the QuantiTect RT Mix, 5 μl of the 2 × QuantiTect Probe PCR Master Mix (Qiagen) and 50 ng total RNA or 1 μL *in vitro *transcript. In minus RT controls the QuantiTect RT Mix was replaced by water. Reverse transcription of one-step RT-PCR was conducted at 50°C for 30 min followed by a 15 min-activation of the HotStartTaq DNA polymerase at 95°C and amplification for 35 cycles (94°C for 20 s, 60°C for 1 min).

### Qualitative detection of A. astaci using qPCR/MCA

The 20-μl duplex qPCR/MCA contained 2 μl 10 × PCR buffer B (Solis BioDyne, Tartu, Estonia), 200 nM of forward and reverse chitinase gene(s) primers (5'-TCAAGCAAAAGCAAAAGGCT and 5'-CCGTGCTCGCGATGGA), 125 nM of forward and reverse 5.8S rRNA primers (5'-ATACAACTTTCAACAGTGGATGTCT and 5'-ATTCTGCAATTCGCATTACG, Figure 6a), 200 μM of each dNTP (Fermentas, St. Leon-Rot, Germany), 0.4 × EvaGreen™ (Biotium), 3.0 mM MgCl_2_, 1 U Taq DNA polymerase chemically modified for "hot start" (Hot FirePol^®^; Solis BioDyne, Tartu, Estonia) and 10 ng DNA template or water in the case of the no-template control. QPCR/MCA was performed on the StepOnePlus™ Real-Time PCR System (Applied Biosystems) run under the StepOne™ software version 2.0. Polymerase activation (95°C for 15 min) was followed by amplification for 35 cycles (95°C for 15 s, 59°C for 15 s and 72°C for 10 s). After an initial denaturation step at 95°C for 15 s, amplicon melting was recorded during a gradual increase of the temperature from 60°C to 95°C.

Oligonucleotides (Sigma-Aldrich, Steinheim, Germany) were designed with Primer Express Software Version 2.0 (Applied Biosystems). The difference between amplicon melting temperatures was calculated using the Nearest Neighbor mode implemented in the online oligonucleotide properties calculator OligoCalc [76].

### Sensitive detection and quantification of A. astaci using TaqMan qPCR

Duplicate TaqMan qPCR was carried out in a total volume of 20 μl containing 2 μl 10 × PCR buffer A2 (Solis BioDyne), 0.2 mM of each dNTP, 4 mM MgCl_2_, 300 nM of each primer (Chi3-324f20 and AaChi-Tmr), 150 nM TaqMan probe (AaChi-FAM), 1 U HOT FIREPol DNA polymerase (Solis BioDyne), 20 ng template DNA or water in the case of the no-template control.

Reactions were amplified in the StepOnePlus™ Real-Time PCR System (Applied Biosystems) under the StepOne™ software version 2.0 using thermal cycling conditions of 15 min at 95°C, followed by 50 cycles of 15 s at 95°C and 1 min at 64°C. A standard curve was generated by plotting the logarithm of the standards copy numbers versus measured *C*_*T *_values.

#### Isolation of spike-in DNA for use in serial dilutions

A crayfish sample extracted from the abdomen of *Cherax quadricarinatus *(Australian red-claw crayfish) was transferred to a 2 ml-extraction tube containing 0.7 g Precellys^® ^ceramic beads of 1.4 mm diameter (Peqlab Biotechnology, Erlangen, Germany) and 180 μl buffer ATL, the lysis buffer of the DNeasy^® ^Blood & Tissue Kit (Qiagen). The MagNA Lyser (Roche) was used for three mechanical lysis cycles consisting of 30 s at 6,500 rpm followed by 60 s on a cooling block held at 4°C. Further isolation was performed according to the protocol "Purification of Total DNA from Animal Tissues (Spin-Column Protocol)" provided by the manufacturer. DNA concentration was determined spectrophotometrically using the Hellma^® ^TrayCell (Hellma, Müllheim/Baden, Germany) on the Eppendorf BioPhotometer 6131.

#### Generation of copy standards

A DNA template stock consisting of *CHI1*, *CHI2 *and *CHI3 *sequences was generated as follows. Genomic DNA from chitinase sequences were amplified with the primers Chi3-324f20 (5'-TCAAGCAAAAGCAAAAGGCT) and AaChi-Tmr (5'-TCCGTGCTCGCGATGGA). Amplification was evaluated by the signal generated from the TaqMan^® ^probe AaChi-FAM (5'-FAM-TCAACGTCCACCCGCCAATGG-BHQ-1). Amplification was performed in a total volume of 20 μl containing 2 μl 10 × PCR buffer A2 (Solis BioDyne), 0.2 mM of each dNTP, 4 mM MgCl_2_, 250 nM of each primer, 150 nM TaqMan probe, 1 U HOT FIREPol^® ^DNA polymerase (Solis BioDyne) and 20 ng DNA or water in the case of the no-template control. DNA denaturation and enzyme activation were performed for 15 min at 95°C. DNA was amplified over 50 cycles consisting of 95°C for 15 s, 60°C for 1 min. QPCR was run on StepOnePlus™ Real-Time PCR System (Applied Biosystems) under the StepOne™ software version 2.0.

PCR fragments were purified with the MSB^® ^Spin PCRapace Kit (Invitek, Berlin, Germany).

The copy number of the target template was determined spectrophotometrically using the Hellma^® ^TrayCell (Hellma, Müllheim/Baden, Germany) on the Eppendorf BioPhotometer 6131. Serial dilutions of the target sequence (10^8 ^to 10^2^, 50, 25 and 12.5 copies per 2 μl) prepared in 10 ng/μl *C. quadricarinatus *DNA were used to determine the amplification efficiency and the quantitative detection limit.

### Statistical analysis of expression changes

A univariate one-way analysis of variance (ANOVA) with Scheffè's post-hoc test was used to evaluate the significance of changes in temporal mRNA expression. The dependent variable was the log-transformed mRNA amount. The time was considered a fixed effect. A value of p < 0.05 calculated by the Scheffè's post-hoc test was regarded as significant. The normality assumption was tested using the Kolmogoroff-Smirnow test.

## Competing interests

The authors declare that they have no competing interests.

## Authors' contributions

GH, RB-R and RS conceived and designed the experiments. RB-R performed Western blot analysis, GH, RT and RS provided the diagnostic assays. GH performed all other experiments. RS supervised the experimental work and the interpretation of data and planned the manuscript. EL provided funding. GH and RS wrote the paper. All authors analysed the data, commented on and approved the manuscript.

## Supplementary Material

Additional file 1 **Species identification of Austrian *A. astaci *strains Gb04, Z12, and GKS07 based on phylogenetic analysis and constitutive chitinase activity in substrate-free medium**. ITS sequence and chitinase expression in chitin-free medium are criteria to classify a strain as *A. astaci* Click here for file

Additional file 2 **Sequences of 3' untranslated regions (UTRs) of *CHI2 *and *CHI3 *mRNAs**. Alignment shows differences between 3' UTRs of CHI2 and CHI3 mRNAs Click here for file

Additional file 3 **Amino-acid substitutions in the GH18 catalytic site of oomycete species**. Table lists amino-acid substitutions in the GH18 catalytic site of oomycete species Click here for file

Additional file 4 **O-linked glycosylation and phosphorylation predicted for Chi2 and Chi3**. Predicted O-linked glycosylations and phosporylations at serine and threonine residues for Chi2 and Chi3 are listed in a table Click here for file

Additional file 5 **Alignment of primer target sites for the *5.8S rRNA *gene used as endogenous control in qPCR/MCA**. Primers target conserved sites in the *5.8S rRNA *gene of various oomycete species Click here for file

Additional file 6 **A conventional PCR assay for detection of *A. astaci *that may fail to discriminate between closely related species**. Alignment of primer sites for a conventional PCR assay reported for detection of *A. astaci* Click here for file

Additional file 7 **Design of a homologous IPC for use in the qPCR/MCA or qPCR assays**. The IPC monitored by a characteristic melting temperature or by an alternatively labeled hydrolysis probe in the qPCR/MCA or qPCR assays, respectively, helps to prevent false-negative detection due to insufficient extraction and/or amplification. Click here for file

Additional file 8 **TaqMan qPCR assay design for sensitive detection and quantification of *A. astaci***. Primers, but also TaqMan probe facilitate discrimination between *A. astaci *and various related or relevant oomycete species. Click here for file
