# Supplementary material for: Identification of two GH18 chitinase family genes and their use as targets for detection of the crayfish-plague oomycete Aphanomyces astaci
Source: BMC Microbiol. 2009 Aug 31;9:184. doi: 10.1186/1471-2180-9-184 (PMC2751781; doi:10.1186/1471-2180-9-184)
Supplement: Additional file 1 — Species identification of Austrian A. astaci strains Gb04, Z12, and GKS07 based on phylogenetic analysis and constitutive chitinase activity in substrate-free medium. ITS sequence and chitinase expression in chitin-free medium are criteria to classify a strain as A. astaci [file 1471-2180-9-184-S2.pdf]

***CHI2*** **TAA**ATGCACATTGAATTCTTTTATATTAGTAACTCATATAACCTCGAATTGACCATTGGGT-TGTG-TGTAAAAAAAAAAAAAAAA
***CHI3*** **TAA**AAGCGCATTCG-TT-GTGTATGTTAGT----CGAATAACCTTGAATTGAAGATTCGATGTGAGTTGTGAAAAAAAAAAAAAAA

**Additional file 2. Sequences of 3' untranslated regions (UTRs) of *CHI2* and *CHI3* mRNAs.** Bold letters: stop codon, grey background: sequence identity, dash: deletion, underline: putative polyadenylation signal. Sequences were derived from GenBank:FJ439177 and FJ386997, respectively.
