# Supplementary material for: Identification of two GH18 chitinase family genes and their use as targets for detection of the crayfish-plague oomycete Aphanomyces astaci
Source: BMC Microbiol. 2009 Aug 31;9:184. doi: 10.1186/1471-2180-9-184 (PMC2751781; doi:10.1186/1471-2180-9-184)
Supplement: Additional file 3 — Amino-acid substitutions in the GH18 catalytic site of oomycete species. Table lists amino-acid substitutions in the GH18 catalytic site of oomycete species [file 1471-2180-9-184-S3.pdf]

Additional file 3. Amino-acid substitutions in the GH18 catalytic site of oomycete species

Species	Protein	GenBank accession number	Conserved consensus sequence (N-terminal → C-terminal)								
			(LIVMFY)	(DN)	G	(LIVMFY)	(DN)	(LIVMFY)	(DN)	X	E
Aphanomyces astaci	Chi2	ABK59977			F						
A. astaci	Chi3	ACJ66260			F						
A. helicoides	Chi1	AAX59706			F						
A. laevis	Chi1	AAX59705			F						
A. repetans	Chi1	ACB12070			F						
Achlya racemosa	Chi1	AAX59707			F						
Leptolegnia caudata	Chi1	AAX59708			F						
Saprolegnia parasitica	Chi1	AAX59704			F						
Trichosporon cutaneum	Chi1	AAX59703			F						

blue: oomycetes; grey: fungus
