# Supplementary material for: Identification of two GH18 chitinase family genes and their use as targets for detection of the crayfish-plague oomycete Aphanomyces astaci
Source: BMC Microbiol. 2009 Aug 31;9:184. doi: 10.1186/1471-2180-9-184 (PMC2751781; doi:10.1186/1471-2180-9-184)
Supplement: Additional file 4 — O-linked glycosylation and phosphorylation predicted for Chi2 and Chi3. Predicted O-linked glycosylations and phosporylations at serine and threonine residues for Chi2 and Chi3 are listed in a table [file 1471-2180-9-184-S4.pdf]

Additional file 4. Phosphorylation and O-linked glycosylation predicted for Chi2 and Chi3. O-glycosylation at residues that would otherwise be phosphorylated is one illustration of the complex interplay among eukaryotic post-translational modification systems (Caragea C, Sinapov J, Silvescu A, Dobbs D, Honavar V: Glycosylation site prediction using ensembles of Support Vector Machine classifiers. *BMC Bioinformatics* 2007, **8**:438.)
Thresholds of > 0.5 and ≥ 0.4 for potential phosphorylated and O-linked glycosylated sites, respectively. Sites for which both types of modification were predicted are highlighted in yellow. Bold letters: modified residue.

position in Chi2	site	score	
		phosphorylation	O-glycosylation
15	VVAM**T**SSVE	0.67	no
16	VAMT**S**SVEA	0.54	no
17	AMTS**S**VEAA	0.60	no
71	VHDG**Y**AAVQ	0.85	no
93	GNNL**Y**GNFG	0.85	no
84	GRGD**S**WNDQ	1.00	no
109	KARG**T**KFGL	0.94	no
114	KFGL**S**IGGW	0.79	no
121	GWTL**S**DKFS	0.51	no
125	SDKF**S**SIAS	0.55	no
126	DKFS**S**IAST	0.83	no
129	SSIA**S**TETG	0.98	no
132	ASTE**T**GRRT	0.94	no
140	TFAK**S**SVKL	0.73	no
141	FAKS**S**VKLM	0.85	no
204	LSVA**S**PAGP	0.92	no
236	DLAG**S**WSK	0.57	no
240	SWSK**Y**TDHQ	0.97	no
248	QANL**Y**EDPN	0.94	no
294	SNGL**Y**SNFT	0.96	no
329	ATEI**Y**DEKL	0.76	no
342	SYDP**T**SKIF	0.62	no
343	YDPT**S**KIFT	0.98	no
347	SKIF**T**SYEG	0.76	no
348	KIFT**S**YEGP	0.98	no
361	QKLD**Y**IKQY	0.72	no
381	ADAK**S**GSPR	0.76	no
383	AKSG**S**PRSL	0.99	no
386	GSPR**S**LITQ	0.77	no
414	PTSQ**Y**ANIR	0.91	no
424	GAAV**T**SAVP	no	0.50
425	AAVT**S**AVPV	no	0.73
430	AVPV**T**SSPV	no	0.88
431	VPVT**S**SPVA	no	0.72
438	VAPV**T**TVAP	no	0.48
439	APVT**T**VAPV	0.69	0.75
444	VAPV**T**SEVP	no	0.69
445	APVT**S**EVPV	no	0.97
450	EVPV**T**SSPA	no	0.81
451	VPVT**S**SPAA	no	0.68
458	AAPV**T**TVAP	no	0.65

459	APVT**T**VAPV	0.69	0.81
464	VAPV**T**SEVP	no	0.85
465	APVT**S**EVPV	no	0.97
470	EVPV**T**SSPD	no	0.65
471	VPVT**S**SPDA	0.99	0.50
472	PVTS**S**PDAP	0.58	no
478	DAPV**T**TVAP	no	0.50
479	APVT**T**VAPV	0.69	0.75
484	VAPV**T**TVAP	no	0.77
485	APVT**S**EVPV	no	0.97
490	EVPV**T**SSPD	no	0.65
491	VPVT**S**SPDA	0.99	0.50
492	PVTS**S**PDAP	0.58	no
498	DAPV**T**TVAP	no	0.56
499	APVT**T**VAPV	0.69	0.80
504	VAPV**T**SAVH	no	0.79
505	APVT**S**AVHV	no	0.83
510	AVHV**T**CSSY	no	0.68
514	TCSS**Y**APVT	0.66	no
518	YAPV**T**SSAV	no	0.45
519	APVT**S**SAVP	no	0.53
525	AVPE**T**TPVE	no	0.67
526	VPET**T**PVEP	0.99	0.52
536	TTEA**T**PAPT	0.92	0.76
532	VEPV**T**TEAT	no	0.84
533	EPVT**T**EATP	no	0.75
540	TPAP**T**GGPI	no	0.85
550	NPLE**T**LAPT	0.55	0.67
554	TLAP**T**TTAA	no	0.40
556	APTT**T**AAAG	no	0.48

position in Chi3	site	score	
		phosphorylation	O-glycosylation
15	VVAM**T**SSVE	0.67	no
16	VAMT**S**SVEA	0.54	no
17	AMTS**S**VEAA	0.60	no
84	EHGD**S**WNDQ	1.00	no
93	GNSL**Y**GNFG	0.86	no
109	KARG**T**KFGL	0.94	no
114	KFGL**S**IGGW	0.79	no
129	SSIA**S**TETG	0.98	no
132	ASTE**T**GRRT	0.94	no
140	TFAK**S**SVKL	0.73	no
141	FAKS**S**VKLM	0.85	no
204	LSVA**S**PAGP	0.92	no
236	DLAG**S**WSK	0.57	no
240	SWSK**Y**TDHQ	0.97	no
248	QANL**Y**EDPN	0.94	no
259	PGAK**Y**SSHN	0.76	no
268	AVQD**Y**IKGG	0.52	no
296	SNGL**Y**SNFT	0.96	no
332	ATEI**Y**DEKL	0.76	no
345	SYDP**T**SKIF	0.62	no
346	YDPT**S**KIFT	0.98	no
350	SKIF**T**SYEG	0.76	no
351	KIFT**S**YEGP	0.98	no
364	QKLD**Y**IKQY	0.72	no
384	ADAK**S**GSPR	0.76	no
386	AKSG**S**PRSL	0.99	no
389	GSPR**S**LITQ	0.77	no
417	PTSQ**Y**ANIR	0.91	no
426	AGAV**T**TVAP	no	0.57
427	GAVT**T**VAPV	0.78	0.51
432	VAPV**T**SAAP	no	0.92
433	APVT**S**AAPV	no	0.80
438	AAPV**T**SAAP	no	0.93
439	APVT**S**AAPV	no	0.99
444	AAPV**T**SSTA	no	0.95
445	APVT**S**STAP	no	0.93
446	PVTS**S**TAPG	no	0.58
447	VTSS**T**APGT	no	0.73
451	TAPG**T**TPAT	no	0.90
452	APGT**T**PATP	0.51	0.89
455	TTPA**T**PVTT	0.68	0.88
458	ATPV**T**TRAT	0.80	0.97
459	TPVT**T**RATP	no	0.85
462	TTRA**T**PAPT	0.97	0.84
466	TPAP**T**GGPI	no	0.90
471	GGPI**T**NPPV	no	0.52
476	NPPV**T**SAPT	0.70	0.96
477	PPVT**S**APTT	no	0.41
480	TSAP**T**TTAA	no	0.64
481	SAPT**T**TAAA	0.52	no
482	APTT**T**AAAG	no	0.58
508	TLPY**S**QADC	0.77	no
