# Supplementary material for: Identification of two GH18 chitinase family genes and their use as targets for detection of the crayfish-plague oomycete Aphanomyces astaci
Source: BMC Microbiol. 2009 Aug 31;9:184. doi: 10.1186/1471-2180-9-184 (PMC2751781; doi:10.1186/1471-2180-9-184)
Supplement: Additional file 5 — Alignment of primer target sites for the 5.8S rRNA gene used as endogenous control in qPCR/MCA. Primers target conserved sites in the 5.8S rRNA gene of various oomycete species [file 1471-2180-9-184-S5.pdf]

A. astaci **(AY310501)**	**ATACAACTTTCAACAGTGGATGTCT**	**CGTAATGCGAATTGCAGAAT**
A. astaci GB04* (EU477365)	....	....
A. astaci GKS07*	....	....
A. astaci Z12*	....	....
A. astaci Hoe (AY683893)	..................N....N.	....
A. astaci Kv1 (AY683894)	....	....
A. astaci Pc (AY683896)	....	....
A. frigidophilus (AY647192)	....	....
A. frigidophilus SAP472 (FM992370)	....	....
A. invadans (FM999229)	....	....
A. invadans WIC (AF396684)	....	....
A. piscicida NJM0003 (AY283641)	....	....
A. sp LIT7 (FM955258)	....	....
A. repetans (AY683897)	....	....
A. repetans LK29 (EU477367)	....	....
A. laevis (FM999237)	....	....
A. stellatus (AY683888)	....	.N..................
A. helicoides (AY310496)	....	....
A. euteiches (FM999226)	....	....
A. cladogamus SAP355 (FM999228)	....	....
A. cochlioides (AY353911)	....	....
A. iridis (FM999227)	....	....
Leptolegnia sp. CBS177.86 (AY310502)	....	....
Leptolegnia sp. K08 (EU240098)	....	....
Leptolegnia sp. PSCR0503 (EU071706)	....	....
Leptolegnia sp. SAP248 (AM228851)	....	....
Achlya racemosa (AF218158)	....	....

Additional file 5.

Alignment of primer sites for the endogenous control *5.8S rRNA* amplicon used in qPCR/MCA. Asterisks denote *A. astaci*-strains isolated in this work. Parentheses contain GenBank accessions. Oomycete reference strains [1] are given in bold. Dot: identical nucleotide with the *A. astaci* sequence.

Reference: 1. Dieguez-Uribeondo J, Garcia MA, Cerenius L, Kozubikova E, Ballesteros I, Windels C, Weiland J, Kator H, Soderhall K, Martin MP: **Phylogenetic relationships among plant and animal parasites, and saprotrophs in Aphanomyces (Oomycetes)**. *Fungal Genet Biol* 2009, **46**(5):365-376.
