# Supplementary material for: Identification of two GH18 chitinase family genes and their use as targets for detection of the crayfish-plague oomycete Aphanomyces astaci
Source: BMC Microbiol. 2009 Aug 31;9:184. doi: 10.1186/1471-2180-9-184 (PMC2751781; doi:10.1186/1471-2180-9-184)
Supplement: Additional file 6 — A conventional PCR assay for detection of A. astaci that may fail to discriminate between closely related species. Alignment of primer sites for a conventional PCR assay reported for detection of A. astaci [file 1471-2180-9-184-S6.pdf]

	525 →	← 640
A. astaci **(AY310501)**	AAGAAGGCTAAATTGCGGTA	CAGAAT-----GCGGAGTCGGATAG
A. frigidophilus (AY647192)	.G.....A............	..A...............GA.....
A. frigidophilus SAP472 (FM992370)	.G.....A............	..A...............GA.....
A. invadans (FM999229)	.G....AA............	..A...............GA.....
A. invadans WIC (AF396684)	.G....AA............	..A...............GA.....
A. invadans NJM9701 (EU422990)	.G....AA............	..A...............GA.....
A. repetans (AY683897)	.G.G..AT............	TGA........-A....CAA..GCT
A. laevis CBS107.52 (AY310497)	.G.G..AT............	TGA........A..AGCAA.AGCCT
A. sp. LIT7 (FM955258)	.G.....A............	....--.....--------------
A. stellatus (AY683888)	.G....AA..........Y.	..NG...........GT.TT.....
A. helicoides (AY310496)	...G..ATG...........	TGA........A-....CAA..G..
A. euteiches (AY647190)	...T..A......G......	TGT.T.TATGGAA.AG.AAA..GCA
A. cladogamus AP355 (FM999228)	...T..AA.....G......	TGTGT.AATGGAA.AG.AGAACGCA
A. cochlioides (AY647191)	.G.G..AT............	TGA........AGA.GCAA.AGCTA
Achlya racemosa (AF218158)	.G...AAA............	..............TT..TAA...A
Leptolegnia sp. SAP248 (AM228851)	.GA...AGC....C......	..AG..........A.A.AAA..TA

Additional file 6.

A conventional PCR assay for detection of *A. astaci* that may fail to discriminate between closely related species.
Alignment of target sites for the *ITS2*-primers 525 (5'-AAGAAGGCTAAATTGCGGTA) and 640 (5'-CTATCCGACTCCGCATTCTG) from [1]. Oomycete reference strains [2] are given in bold. Dot: identical nucleotide with the *A. astaci* sequence; gap: deletion.

References

1. Oidtmann B, Schaefers N, Cerenius L, Söderhäll K, Hoffmann RW: **Detection of genomic DNA of the crayfish plague fungus Aphanomyces astaci (Oomycete) in clinical samples by PCR**. *Vet Microbiol* 2004, **100**(3-4):269-282.
2. Dieguez-Uribeondo J, Garcia MA, Cerenius L, Kozubikova E, Ballesteros I, Windels C, Weiland J, Kator H, Soderhall K, Martin MP: **Phylogenetic relationships among plant and animal parasites, and saprotrophs in Aphanomyces (Oomycetes)**. *Fungal Genet Biol* 2009, **46**(5):365-376.
