# Supplementary material for: Identification of two GH18 chitinase family genes and their use as targets for detection of the crayfish-plague oomycete Aphanomyces astaci
Source: BMC Microbiol. 2009 Aug 31;9:184. doi: 10.1186/1471-2180-9-184 (PMC2751781; doi:10.1186/1471-2180-9-184)
Supplement: Additional file 7 — Design of a homologous IPC for use in the qPCR/MCA or qPCR assays. The IPC monitored by a characteristic melting temperature or by an alternatively labeled hydrolysis probe in the qPCR/MCA or qPCR assays, respectively, helps to prevent false-negative detection due to insufficient extraction and/or amplification. [file 1471-2180-9-184-S7.pdf]

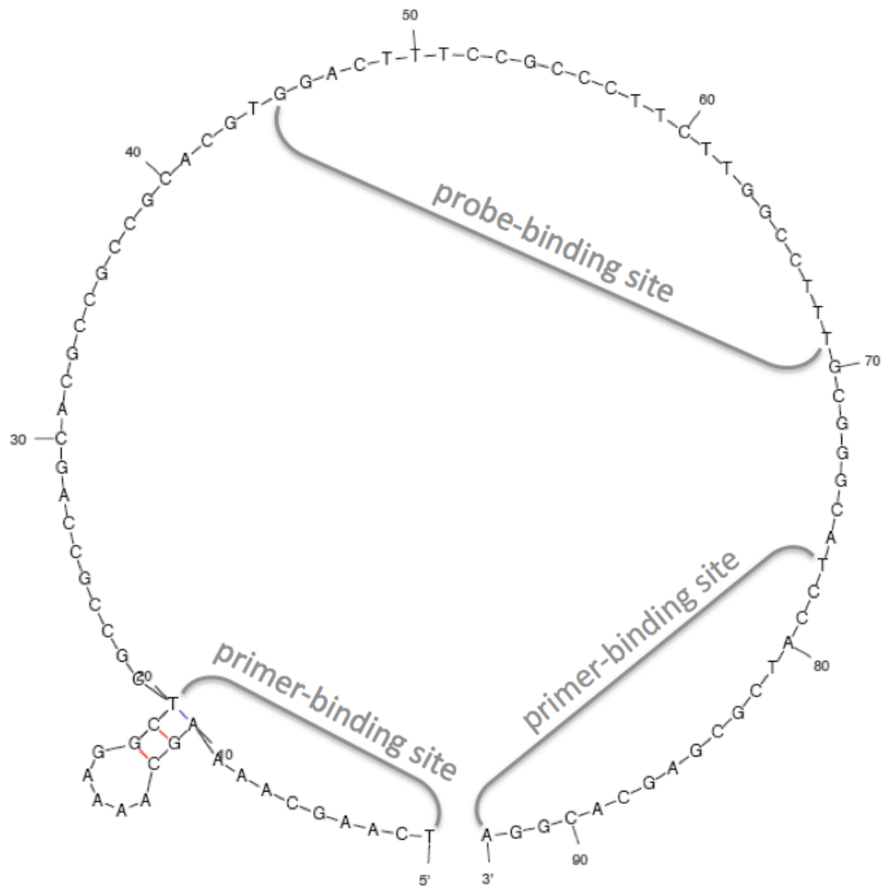

Additional file 7.
Design of a homologous IPC for use in the qPCR/MCA or qPCR assays. This DNA control can be used for coextraction and/or coamplification with the clinical samples. The Mfold web server [1] was used for prediction of its secondary structure ($\Delta G = -0.15$ kcal/mol).
The 93 bp sequence (TCAAGCAAAAGCAAAAGGCTCGCCGCCAGCACGCCGCCGCACGT **GGACTTTCCGCCCTTCTTGGCCTTT**GCGGGCATCCATCGCGAGCACGGA) is compatible with the qPCR/MCA and the TaqMan qPCR assays developed in this work. The target sites of the primers used in the qPCR/MCA and the qPCR assays for *A. astaci*-detection are underlined. Note that the 16 bp-reverse primer used in qPCR/MCA is shortened by a base at its 5' end compared to the reverse primer of TaqMan qPCR. Bold letters denote the site targeted by the hydrolysis probe DYE-AAAGGCCAAGAAGGGCGGAAAGTCC-BHQ to monitor the IPC in the TaqMan qPCR assay designed for *A. astaci*-quantification.
In the dye-based qPCR/MCA assay the IPC can be differentiated by a 3 °C higher melting temperature from the *A. astaci*-specific chitinase peak. This was predicted by the Nearest Neighbour method implemented in the online oligonucleotide properties calculator OligoCalc [2].

References:
1. Zuker M: **Mfold web server for nucleic acid folding and hybridization prediction**. *Nucleic Acids Res* 2003, **31**(13):3406-3415.
2. Kibbe WA: **OligoCalc: an online oligonucleotide properties calculator**. *Nucleic Acids Res* 2007, **35**(Web Server issue):W43-46.
