# Supplementary material for: Identification of two GH18 chitinase family genes and their use as targets for detection of the crayfish-plague oomycete Aphanomyces astaci
Source: BMC Microbiol. 2009 Aug 31;9:184. doi: 10.1186/1471-2180-9-184 (PMC2751781; doi:10.1186/1471-2180-9-184)
Supplement: Additional file 8 — TaqMan qPCR assay design for sensitive detection and quantification of A. astaci. Primers, but also TaqMan probe facilitate discrimination between A. astaci and various related or relevant oomycete species. [file 1471-2180-9-184-S8.pdf]

A. astaci CHI3 (FJ457089)	**TCAAGCAAAAGCAAAAGGCT**	**CCATTGGCGGGTGGACGTTGA**	**TCCATCGCGAGCACGGA**
A. astaci CHI2 (DQ974157)	....	....	..............A..
A. astaci CHI1 (AY866375)	....	....	....
A. frigidophilus* SAP472, *CHI1 (GQ152605)	.............GCGC..C	.G..C............C...	GG...............
A. frigidophilus* NJM 9665, *CHI1 (GQ152604)	.............GCGC..C	.G..C............C...	GA...............
A. invadans CHI1 (GQ374536)	A.....TC...A.....TTC	.......T.............	GA......CGA.T....
A. laevis CHI1 (AY866379)	A.....TC...A.GC..TTC	....C..T..C.....TC.C.	GG......C.....C..
A. repetans CHI1 (EU523123)	A.....TC...A.GC..TTC	....C.....A.....CC.C.	GG......C.....C..
A. helicoides CHI1 (AY866380)	A.....TC...A.GC..TTC	....C.....C.....CC.C.	AG......C.....C..
Leptolegnia caudata CHI1 (AY866382)	A....ATG...A.G...TT.	.G..C.....C..........	GG.T.T..C.....C..
Saprolegnia parasitica CHI1 (AY866378)	A....ATG...A.GGTCCA.	.G..C..T..C......C.C.	GG.T.T..AGC...C..
Achlya racemosa CHI1 (AY866381)	AT...ATG..AA.G...TAC	.T..C.....T......C.C.	GGGT.TT.T........
Trichosporon cutaneum CHI1 (AY866377)	A....ATG..AA.G.C.CAC	.G..C.....C......C.C.	GG.T.T...GCG..C..
Fusarium solani CHI1 (AY866376)	A.CTC.TC...AGGCGCAAC	.AG.C..A..A.....C.AC.	G.TCC...T.....CCC

Additional file 7.
TaqMan qPCR assay design for sensitive detection and quantification of *A. astaci*.
Target sites of primers and probe are highlighted by arrows and line, respectively. Parentheses contain GenBank accession numbers. Primers of the TaqMan qPCR and qPCR/MCA assays are identical except the deleted nucleotide at the very 5’ end of the reverse primer used in qPCR/MCA (Figure 5a).
